# Fatty acid and cholesterol profiles, hypocholesterolemic, atherogenic, and thrombogenic indices of broiler meat in the retail market

**DOI:** 10.1186/s12944-017-0423-8

**Published:** 2017-02-16

**Authors:** Youssef A. Attia, Mohammed A. Al-Harthi, Mohamed A. Korish, Mohamed M. Shiboob

**Affiliations:** 1 Arid Land Agriculture Department, Faculty of Meteorology, Environment and Arid Land Agriculture, King Abdulaziz University, P.O. Box 80208, Jeddah, 21589 Saudi Arabia; 2 Enviromental Department, Faculty of Meteorology, Environment and Arid Land Agriculture, King Abdulaziz University, P.O. Box 80208, Jeddah, 21589 Saudi Arabia; 3 Department of Food and Dairy Science &Technology, Faculty of Agriculture, Damanhour University, Damanhour, 22516 Egypt

**Keywords:** Atherogenic, Fatty acids, Hyper*cholesterolemia*, Thrombogenic, Broiler meat

## Abstract

**Background:**

Broiler meat is an essential source of food due to its favourable effects on human health derived from its protein, fats, minerals, vitamins and its bioactive components.

**Methods:**

A total of 90 carcasses were collected from the retail market in Jeddah city, Saudi Arabia during April, May and June 2014 to determine the effects of meat type (frozen vs. fresh) and sources within fresh types (A, B, C) vs. frozen types (D, E and F) on their fatty acid profiles, cholesterol, their hypocholesterolemic, atherogenic and thrombogenic indices, and on their antioxidants’ status.

**Results:**

The sources of meat had a significant effect on the hypocholesterolemic and atherogenic indices, with the D source of fresh meat having the best indices. Total saturated fatty acids (SFA), unsaturated fatty acids (UFA), the UFA/SAF ratio, and the monounsaturated (MUFA), Omega-6 and Omega-7 fatty acids were significantly affected by the source of meat. The results revealed that the D source of fresh meat showed favourable fatty acid profiles with significant health benefits for human. Correlation analyses showed a significant negative relationship between the SFA and hypocholesterolemic indices, and significant positive relationships with the atherogenic index, the thrombotgenic index and the total antioxidant capacity. In addition, the relationship between UFA and the hypocholesterolemic index was strongly significantly positive, but was highly negative between the atherogenic and thrombotic indices. The correlations between omega-6 and total cholesterol and the atherogenic index was moderately negative, but was moderately positive with the hypocholesterolemic index.

**Conclusion:**

Fatty acids profiles and the hypocholesterolemic and atherogenic indices of broiler meat in the retail market in Jeddah city, Saudi Arabia during April-May-June showed significant differences, with the potential for favourable fatty acids to be boosted. Such variability indicates the needs for a feeding strategy to enhance the favourable fatty acids that may positively impact the health of the consumer, lowering the risk of hypercholesterolemia, atherosclerosis, and thrombogenesis although further studies are needed.

## Background

Chicken meat is one of the major animal protein supplies. It contains all the nutrients that can meet the recommended daily allowances for humans [1]. Poultry meat is an extremely complex item can be assessed from the standpoint of consumer interests and from that of the slaughter industry [2]. Chicken meat has a relatively low fat content and is a respected source of proteins, vitamins and mineral sources [3].

Consumption of foods that rich of saturated fat and cholesterol, especially from animal sources were found to be responsible for the development of coronary heart diseases [4]. Ecological studies across 16 defined populations in seven countries revealed that intake of saturated fat as a percentage of calories had strong correlation with coronary death rates [5], where each 5% increase of energy from saturated fat was associated with 17% increase in coronary heart diseases [6]. Poultry meat contains a low proportion of fat and cholesterol, where it contain around 1% fat in the leanest cuts, and 17% in cooked chicken wings with skin. Poultry fat is favorable: it includes significant amounts of monounsaturated fatty acids (only a third of total fat is made up of saturated fatty acids and, in comparison with red meat, substantial amounts of polyunsaturated fats, especially the omega-6 or n-6 linoleic acid and arachidonic acid [7]. It may represent an important source of long-chain omega-3 or n-3 fatty acids [8]. In the California Seventh-day Adventist study, higher consumption of poultry was not associated with risk of coronary heart diseases [9]. A very large observational study carried out in the United States on a female population reported an inverse relationship between the intakes of poultry and the risk of developing coronary artery disease [10].

The chemical composition of muscles is an important quality measurement of broiler meat [11]. Nutrients profiles, such as fat/fatty acids and the hypocholesterolemic, atherogenic and thrombotic indices of poultry products, were found to be important concepts in meat consumption [2, 12–15]. Meat quality is a complex concept that is influenced by the strain of broilers [2, 16, 17] and by diet composition, feed types and additives [13, 18, 19]. Additional influences are rearing practices, the health of the chickens, the environmental conditions, slaughter practices, storage, handling and cooking [20–22] as well as fresh vs. frozen meat [23, 24].

The lipids and antioxidants of poultry product are essential components from the health and consumption perspectives for humans. This status has considerable impact on meat consumption and its health implications [25–28]. Therefore studying the variability in the profiles of its fats/fatty acid profiles, cholesterol, its hypocholesterolemic, atherogenic and thrombotic indices, and its antioxidants and in its lipid peroxidation biomarkers, such as malnodialdehyde (MDA), is essential for developing quality control and for mentoring the quality of broiler meat in the retail market and its impact on human health.

## Results

### Cholesterol profiles, atherogenic and thrombotic indices, and antioxidant status

Table 1 shows the effects of different meat types (frozen vs. fresh) and meat sources (A, B, C, D, E and F) on the cholesterol profiles, atherogenic and thrombotic indices, and antioxidant status of meat. Frozen meat had a numerically (*P* = 0.054) higher hypocholesterolemic index, but a significantly lower (*P* = 0.05) thrombotic index when compared to fresh meat. On the other hand, differences in the lipid profiles and antioxidant indices were not significant between the types and sources of meat, except for the hypocholesterolemic and atherogenic indices, which were significantly affected by the source of meat only. Source D of fresh meat had a significantly greater hypocholesterolemic index than the other sources, except for sources A and B of frozen meat. Also, source A of frozen meat had a significantly higher hypocholesterolemic index than sources E and F of fresh meat. In addition, sources B and C of frozen meat had significantly higher hypocholesterolemic indices than did source E of fresh meat.

**Table 1:** Effect of meat type and different source of frozen and fresh meat in the retail market on cholesterol profiles, and hypocholesterolemic, atherogenic and thrombotic indices, different type of cholesterol and indices of antioxidant status of fresh meat

Parameters	Total cholesterol, mg/100 g	Low density lipoprotein, mg/100 g	High density lipoprotein, mg/100 g	LDL/HDL ratio	Hypocholesterolemic index	Atherogenic index	Thrombotic index	TAC mmoles/100 g fresh meat	MDA, mmoles/g fresh meat
Meat type									
Frozen meat	73.1	37.7	40.6	0.931	1.91	0.726	0.679	1.50	1.95
Fresh meat	72.4	36.9	39.6	0.933	1.77	0.782	0.697	1.51	1.84
Source of meat									
Frozen A	73.2	37.4	40.8	0.916	2.01^ab^	0.650^c^	0.656	1.54	1.92
Frozen B	72.4	38.3	39.7	0.966	1.89^abc^	0.722^bc^	0.671	1.45	1.96
Frozen C	73.7	37.4	41.3	0.908	1.83^bc^	0.807^ab^	0.709	1.52	1.95
Fresh D	70.5	36.7	39.2	0.938	2.11^a^	0.658^c^	0.664	1.32	1.69
Fresh E	73.7	36.8	40.1	0.920	1.55^d^	0.891^a^	0.722	1.66	1.98
Fresh F	73.1	37.2	39.4	0.942	1.66^cd^	0.795^ab^	0.704	1.54	1.86
Statistical analyses									
RSME	2.56	1.905	1.725	0.067	0.186	0.037	0.134	0.204	0.218
Meat type	0.466	0.258	0.126	0.915	0.054	0.185	0.007	0.958	0.207
Source of meat	0.302	0.922	0.594	0.666	0.0006	0.036	0.201	0.134	0.383

^a-d^Differences among means within a column within each factor not sharing similar superscripts are significantly at (*P* <0.05); *P value* probability level, *RMSE* root mean square error, *TAC* total antioxidant capacity, *MDA* melondialdehyde

Source A of frozen meat and source D of fresh meat had significantly lower atherogenic indices than the other sources, except for source B of frozen meat.

### Fatty acid profiles of meat

Tables 3 and 4 show the effects of the meat types and meat sources on individual and total fatty acid profiles. There was a significant effect of meat type on individual SFA (Table 2) and total SFA (Table 3). Fresh meat showed significantly greater C8:0, C11:0, C12:0, and C16:0, and thus total SFA, than did frozen meat. There was also a significant effect of meat source on individual SFA (Table 2) and total SFA (Table 3). Source E of fresh meat had significantly greater C8:0, C11:0, C12:0 and C16:0 than did the other sources of frozen and fresh meat. A similar trend was observed for C21:0, except that the differences between sources E and F of fresh meat was insignificant. This latter group had significant greater C21:0 than its counterpart group from source D. The total SFA of fresh meat from source E was significantly greater than from that of the other sources, except for source F of fresh meat. The latter group, and source C of frozen meat, had greater total SFA than did source A of frozen meat and source D of fresh meat.

**Table 2:** Effect of meat type and different source of frozen and fresh meat in the retail market on individual fatty acid profiles (% of total fatty acids) of meat

	Saturated fatty acids (% of total fatty acids)										Monounsaturated fatty acids (% of total fatty acids)							Polyunsaturated fatty acids (% of total fatty acids)			
Parameters	C8:0	C10:0	C11:0	C12:0	C13:0	C14:0	C15:0	C16:0	C17:0	18.0	21:0	C14:1	C15:1	C16:1	C18:1	C20:1	C22:1	C18:2	C18:3	C20:5	C20:4
Meat type																					
Frozen meat	0.049	0.084	0.279	0.663	1.134	2.15	1.012	28.7	0.116	11.9	1.44	0.386	0.414	3.44	22.9	0.435	1.14	23.6	0.043	0.088	0.166
Fresh meat	0.089	0.111	0.538	0.954	0.826	1.96	0.872	30.1	0.124	12.3	1.72	0.372	0.329	3.54	19.0	0.577	1.95	24.4	0.051	0.115	0.144
Source of meat																					
Frozen A	0.043^b^	0.044	0.265^b^	0.551^b^	0.910	1.70	0.643	28.2^b^	0.139	11.2	1.44^bc^	0.262	0.268	3.92	25.5^a^	0.614^ab^	1.05^b^	23.1^b^	0.035	0.056^bc^	0.154
Frozen B	0.055^b^	0.105	0.336^b^	0.633^b^	0.906	1.86	0.835	29.3^b^	0.102	12.5	1.46^bc^	0.297	0.356	3.49	22.5^ab^	0.404^bc^	1.22^b^	23.5^b^	0.052	0.010^c^	0.163
Frozen C	0.051^b^	0.103	0.236^b^	0.804^b^	1.587	2.88	1.555	28.5^b^	0.107	12.0	1.41^bc^	0.599	0.637	2.90	20.6^bc^	0.289^c^	1.14^b^	24.1^b^	0.042	0.197^b^	0.180
Fresh D	0.039^b^	0.060	0.170^b^	0.638^b^	0.921	2.18	0.975	27.1^b^	0.110	11.1	0.93^c^	0.393	0.422	3.55	21.5^b^	0.811^a^	0.616^b^	28.1^a^	0.052	0.346^a^	0.020
Fresh E	0.171^a^	0.062	1.157^a^	1.336^a^	0.756	1.95	0.877	31.8^a^	0.159	13.3	2.30^a^	0.336	0.316	3.52	17.4^c^	0.428^bc^	2.59^a^	21.2^b^	0.056	0.00^c^	0.157
Fresh F	0.057^b^	0.212	0.285^b^	0.887^b^	0.800	1.75	0.764	31.3^a^	0.104	12.5	1.95^ab^	0.387	0.251	3.54	18.0^c^	0.493^bc^	2.64^a^	23.9^b^	0.046	0.00^c^	0.255
Statistical analyses																					
RSME	0.033	0.091	0.184	0.329	0.439	0.691	0.535	1.564	0.078	1.566	0.544	0.259	0.248	0.536	2.523	0.209	0.589	2.481	0.028	0.141	0.146
Meat type	0.004	0.418	0.001	0.0236	0.0662	0.465	0.482	0.021	0.769	0.442	0.160	0.882	0.362	0.614	0.003	0.077	0.001	0.375	0.411	0.601	0.686
Source of meat	0.001	0.055	0.001	0.028	0.115	0.081	0.111	0.001	0.709	0.189	0.009	0.291	0.149	0.091	0.010	0.014	0.001	0.005	0.826	0.001	0.195

^a–c^Differences among means within a column within each factor not sharing similar superscripts are significantly at (*P* <0.05); *P value* probability level, *SFA* saturated fatty acids, *UFA* unsaturated fatty acids, *PUFA* poly unsaturated fatty acids, *MUFA* monounsaturated fatty acids, *RMSE* root mean square error

**Table 3:** Effect of meat type and different source of frozen and fresh meat in the retail market on fatty acid profiles (% of total fatty acids) of meat

Parameters	SFA (%)	UFA (%)	UFA/SFA ratio	Total PUFA	Total PUFA/UFA	MUFA (%)	PUFA/MUFA ratio	Omega-3 PUFA (%)	Omega-6 PUFA (%)	Omega-6/omega -3 ratio	Omega -7	Omega -9
Meat type												
Frozen meat	47.4	52.5	1.12	23.7	0.455	28.7	1.22	0.297	23.6	113.4	28.3	25.1
Fresh meat	49.6	50.4	1.04	24.7	0.488	25.7	1.06	0.311	24.4	182.6	25.4	22.9
Source of meat												
Frozen A	45.1^cd^	54.9^ab^	1.23^ab^	23.4^b^	0.426	31.6^a^	1.36	0.244	23.1^b^	142.1	31.3^a^	27.6
Frozen B	48.0^bc^	51.9^bc^	1.09^bc^	23.7^b^	0.455	28.2^b^	1.22	0.226	23.5^b^	136.5	27.9^b^	24.9
Frozen C	49.2^b^	50.8^c^	1.04^c^	24.6^b^	0.484	26.2^bc^	1.08	0.420	24.1^b^	61.5	25.6^bc^	22.9
Fresh D	44.2^d^	55.8^a^	1.27^a^	28.5^a^	0.511	27.3^b^	0.96	0.419	28.1^a^	130.9	26.8^b^	22.7
Fresh E	53.9^a^	46.0^d^	0.86^d^	21.4^b^	0.466	24.6^c^	1.16	0.212	21.2^b^	248.9	24.3^c^	22.6
Fresh F	50.6^ab^	49.4^cd^	0.98^cd^	24.1^b^	0.487	25.3^b^	1.06	0.301	23.9^b^	167.8	25.0^b^	23.2
Statistical analyses												
RSME	2.75	2.75	0.127	2.56	0.041	2.41	0.192	0.202	2.48	167.4	2.38	2.39
Meat type	0.046	0.046	0.1005	0.389	0.037	0.003	0.035	0.853	0.374	0.269	0.003	0.015
Source of meat	0.001	0.001	0.002	0.004	0.119	0.013	0.124	0.276	0.004	0.732	0.008	0.074

^a-d^Differences among means within a column within each factor not sharing similar superscripts are significantly at (*P* <0.05); *P value* probability level, *SFA* saturated fatty acids, *UFA* unsaturated fatty acids, *PUFA* poly unsaturated fatty acids, *MUFA* monounsaturated fatty acids, *RMSE* root mean square error

There was no significant difference in most of MUFA except for C18:1 and C22:1. The C18:1 fatty acid of frozen meat was significantly greater (20.5%) than that of fresh meat, but the C22:1 fatty acid was lower (41.5%). Total MUFA was significantly greater (11.7%) in frozen meat than in fresh meat. The differences among the different sources of meat were significant in their C18:1, 20:1 and 22:1 fatty acids. Source A of frozen meat had significantly greater C18:1 fatty acids than did the other sources, except for source B of frozen meat. Source B also showed a similar trend, except that it did not significantly differ from source C of frozen meat and source D of fresh meat. In addition, source D of fresh meat had significantly greater C20:1 than did the other sources of both types of meat except for source A of frozen meat. The latter group also exhibited C18:1 that was greater than different sources except for source B of frozen meat. Moreover, sources E and F of frozen meat showed significantly greater C22:1 than did the other groups of either frozen or fresh meat. The total MUFA of source A of frozen meat was significantly greater than that of other sources. Furthermore, source B of frozen meat and sources D and F of fresh meat exhibited greater total MUFA than did source E of fresh meat.

The only significant difference due to the source of meat was shown in C20:5 PUFA (Table 2), which indicates greater values from source D of fresh meat than from other sources. In addition, source C of frozen meat displayed a significantly greater value than did source B of frozen meat and sources E and F of fresh meat. The total PUFA of fresh meat source D was significantly greater than that of other sources (Table 3).

Obviously, the PUFA/UFA ratio was significantly greater (7.3%) in fresh meat than in frozen meat, but PUFA/MUFA (13.1%), omega-7 (10.3%) and omega-9 (8.8%) fatty acids were lower (Table 3). The UFA and UFA/SFA ratio were significantly greater from D source of fresh meat than from the other sources, except for source A of frozen meat. The latter group also showed higher values than did the other groups, except for source B of frozen meat.

Omega-6 was also significantly greater in source D of fresh meat than in the other sources. Omega-7 in frozen meat was significantly greater from source A of frozen meat than from other sources. In addition, sources B of frozen meat and D and F of fresh meat had significantly greater omega-7 than did source E of fresh meat.

### Correlation analyses

Correlation analyses are displayed in (Table 4). There were significant negative relationships between SFA and UFA, MUFA, PUFA, omega-6, omega-9, omega-7 and the hypocholesterolemic index, but significant positive relationships with the atherogenic index, the thrombogenic index and total antioxidant capacity.

**Table 4:** Correlation among indices lipids profiles, antioxidant status, atherogenic and thrombotic index of chickens’ meat in the retail market

	SFA	UFA	MUFA	PUFA	Omg3	Omg6	Omg9	Omg7	Cho	LDL	HDL	AthI	ThrI	HypochI	TAC	MDA
SFA	1.000 0.000	-0.999 0.001	-0.647 0.001	-0.661 0.001	-0.337 0.069	-0.659 0.001	-0.453 0.012	-0.654 0.001	0.379 0.039	-0.049 0.797	0.229 0.222	0.940 0.001	0.605 0.004	-0.907 0.001	0.519 0.003	0.353 0.055
UFA		1.000 0.000	0.654 0.001	0.655 0.001	0.336 0.069	0.653 0.001	0.459 0.011	0.660 0.001	0-.38 0.037	0.047 0.804	-0.227 0.228	-0.94 0.001	-0.604 0.004	0.907 0.001	-0.51 0.003	-0.353 0.056
MUFA			1.000 0.000	-0.143 0.450	-0.090 0.635	-0.144 0.447	0.920 0.001	0.996 0.001	0.036 0.851	0.158 0.402	0.029 0.877	-0.602 0.004	0.546 0.002	0.569 0.001	-0.004 0.985	0.078 0.685
PUFA				1.000 0.000	0.525 0.002	0.998 0.001	-0.318 0.086	-0.131 0.487	-0.53 0.002	-0.099 0.603	-0.319 0.086	-0.627 0.002	-0.248 0.186	0.616 0.003	-0.669 0.001	-0.537 0.002
Omg3					1.00 0.000	0.478 0.008	-0.146 0.440	-0.096 0.612	-0.20 0.302	-0.131 0.489	0.102 0.958	-0.268 0.152	0.521 0.003	0.439 0.015	-0.473 0.008	-0.239 0.202
Omg6						1.000 0.000	-0.320 0.084	-0.131 0.487	-0.54 0.002	-0.093 0.626	-0.334 0.071	-0.629 0.002	-0.287 0.123	0.607 0.004	-0.663 0.001	-0.540 0.002
Omg9							1.00 0.000	0.934 0.001	0.073 0.701	0.035 0.855	0.058 0.761	-0.487 0.006	-0.401 0.028	0.372 0.04	0.086 0.651	0.103 0.586
Omg7								1.000 0.000	0.01 0.959	0.134 0.479	0.013 0.944	-0.631 0.002	-0.553 0.002	0.570 0.001	-0.020 0.915	0.062 0.747
Chol									1.000 0.000	0.59 0.001	0.529 0.003	-0.934 0.001	-0.548 0.002	-0.321 0.082	0.754 0.001	0.753 0.001
LDL										1.00 0.000	-0.095 0.619	-0.056 0.768	0.172 0.364	-0.024 0.90	0.181 0.337	0.400 0.028
HDL											1.000 0.000	0.032 0.869	-0.170 0.371	-0.115 0.544	0.579 0.001	0.592 0.001
AthI												1.000 0.000	0.591 0.006	-0.882 0.0001	0.508 0.004	0.356 0.054
ThrI													1.000 0.000	-0.415 0.022	0.113 0.551	0.156 0.409
HypochI														1.00 0.00	-0.426 0.02	-0.278 0.136
TAC															1.000 0.000	0.754 0.001
MDA																1.000 0.000

*SFA* saturated fatty acids, *UFA* unsaturated fatty acids, *PUFA* poly unsaturated fatty acids, *MUFA* monounsaturated fatty acids, *Omg3* omega 3 fatty acid, *Omg6* omega 6 fatty acid, *Omg7* omega 7 fatty acid, *Omg9* omega 9 fatty acid, *LDL* low density lipoprotein, *HDL* high density lipoprotein, *AthI* atherogenic index, *ThrI* thrombotic index, *HypochI* hypocholesterolemic index

Correlations between UFA and MUFA, PUFA, omega-3, omega-7 were significantly positive with a moderate magnitude, while the correlation between UFA and the hypocholesterolemic index was significantly positive with a strong magnitude. In addition, the correlation between UFA and omega-9 was significant with a moderate magnitude. On the other hand, the correlations between UFA, cholesterol and MDA were significantly negative with a low magnitude; the correlation between UFA and total antioxidant capacity (TAC) was negative to a moderate power, and the correlation between UFA and the atherogenic and thrombogenic indices was significantly negative with a high magnitude.

The relationship between MUFA and omega-7 and omega-9 was significantly positive with a perfect magnitude, while the correlation between MUFA and the atherogenic, thrombotic and hypocholesterolemic indices was significantly positive with a moderate power.

The correlation between PUFA and omega-6 was significantly positive to a perfect power, while the relationship between PUFA, omega-3 and the hypocholesterolemic index was significantly positive to a moderate magnitude. In addition, the correlation between PUFA and total cholesterol, the atherogenic index, TAC and MDA was significantly negative to a moderate magnitude.

The relationship between omega-3 and omega-6 and the thrombotic and hypocholesterolemic indices was significantly positive to a moderate power, but negative to a moderate power with TAC.

The correlation between omega-6 and total cholesterol, the atherogenic index, TAC and MDA was negative to a moderate power, but was positive to a moderate magnitude with the hypocholesterolemic index.

The correlation between omega-9 with omega-7 and the hypocholesterolemic index was significantly positive to perfect and low powers, respectively, but the relationship with the atherogenic index and the thrombotic index was negative with a moderate magnitude.

The relationship between omega-7 and the hypocholesterolemic index was significantly positive with moderate power, while the correlation with the atherogenic index and the thrombogenic index was negative to a moderate magnitude.

There was a significant positive correlation between total cholesterol and LDL- and HDL- cholesterol with a moderate magnitude, while the relationship between total cholesterol and TAC and MDA was positive to a high magnitude. In addition, the correlation between total cholesterol with the atherogenic and thrombogenic indices was significantly negative with perfect and moderate magnitudes, respectively.

LDL cholesterol correlated positively with MDA, but the power of the correlation was moderate, while HDL cholesterol showed a positive significant correlation with a moderate magnitude with TAC and MAD. The atherogenic index exhibited a significant positive moderate relationship with the thrombotic index and with TAC, but the relationship was significantly negative with a high power with the hypocholesterolemic index. The thrombotic index showed a significant negative moderate relationship with the hypocholesterolemic index. The relationship between TAC and MDA was significantly positive with a strong power.

## Discussion

In the literature, the relationship between type of food consumed and particularly dietary fats, antioxidants and health status are well documented [4, 25–28]. Epidemiological studies conducted worldwide, supported the association between poultry meat consumption, within a balanced diet, and a reduction in the risk of developing cardiovascular diseases and their risk factors [29] Other study reveals that, the substitution of red meat with poultry could therefore constitute an effective strategy to reduce coronary risk by 19% [30]. It was observed that differences in the hypocholesterolemic index of meat from broilers from the retail market were found to be affected by the type of meat (frozen vs. fresh). Source D of fresh meat showed the greatest hypocholesterolemic index and the lowest total SFA and MDA, but the highest UFA, UFA/SFA, PUFA and omega-6, which are favourable effects. In the literature, as shown herein, high cholesterol correlates with high SFA content (*r* = -0.907; *P* = 0.001) [4, 15, 31, 32]. Further evidence supported our hypothesis that broiler meat quality in the retail market has different nutritional values, and hence we can assume different human health impacts. This can be seen in differences in the atherogenic index and in the omega-6 fatty acids. Hence the favourable hypocholesterolemic, atherogenic and thrombogenic indices, and the favourable TAC and MDA, were from the fresh meat, indicating that freezing the meat had a negative impact on its quality. These differences can primarily be attributed to diet composition, i.e. to the consumption of the feeds offered to the animals [13, 19, 33]. This indicates that consumers can benefit their health by being selective with broiler meat, not only for improving their PUFA fatty acid intake, but also for reducing their hypocholesterolemic, atherogenic and thromboitic indices. The freezing technique can affect the carcasses and the meat quality, as the freezing process can affect the water fraction of the meat [34]. With the freezing of water, the concentration of the remaining solutes (proteins, carbohydrates, lipids, vitamins and minerals) increases, thereby upsetting the homeostasis of the complex meat system [35]. The changes in the immediate environment of the muscle fibres affect the cell membrane characteristics. That, in turn, affects the quality of the meat [35]. Consumers’ preference for leaner carcasses has also increased in recent years [36].

The quality of poultry meat is affected by many factors, such as dietary nutrient intake. The most biological aspects are genotype, sex and age [2, 27, 33, 37]. Another factor that can influence poultry meat quality and compositional changes in the meat is the rearing system for broilers [2, 38–40]. Hence, increasing intakes of SFA, cholesterol, and the atherogenic and thrombogenic indices have negative health implications [2, 4, 33]. These substances have been connected with higher cardiovascular threats and with thrombosis associated with increasing levels of blood cholesterol [28, 29, 33, 41, 42]. In this regard, the hypocholesterolemic index was found to be significantly correlated with the thrombogenic index and with TAC. This indicates a need for standardization of nutrition in terms of the fatty acid contents of diets and in husbandry practices to minimise variation and improve quality of chickens’ meat.

The lack of significant differences between TAC and MDA (the biomarker for lipid peroxidation) among the different meat types and sources indicates that the intake of antioxidants such as vitamin E, vitamin C, carotenoids and Se was adequate for postharvest preservation. Antioxidants are usually supplemented at sufficient amounts, considering the recent trend in animal nutrition for improving the shelf life of animal products [2, 25, 26], of animal fatty acid profiles and of immunity [2, 33, 43].

Meat fatty acids profiles, SFA, UFA, MUFA, PUFA, omega-3, omega-6, omega-7 and omega-9, and the ratios among them, showed a significant effect of the type or source of meat that can be attributed to dietary composition and feed consumption. In general, source D of fresh meat showed the best profile, showing the lowest SFA, with the highest UFA, UFA/SFA, PUFA, omega-3 and omega-6 PUFA while the worst profile was from source E of fresh meat.

The D source of fresh meat was the richest source of linoleic and of eicosapentaenoic acid (EPA). EPA has been reported as a fatty acid that is beneficial for reductions in total mortality, cardiovascular mortality, morbidity, atherosclerosis [44], for obstructing the propagation of breast and prostate cancer cell lines in vitro, and for reducing the risk and development of these tumours in animal experiments [45]. It should be mentioned that the conversion of α-linolenic acid by the body to the more active longer-chain metabolites is inefficient, being 5–10% for EPA and 2–5% for DHA [46]. A common feature of most of the proposed mechanisms by which fatty acids might lower cancer risk is the inhibition of eicosanoid production from (n-6) fatty acid precursors [47], which include linoleic acid, found primarily in vegetable oils, and arachidonic acid, found primly in animal products.

Source A of frozen meat showed a fatty acid profile similar to that of source D of fresh meat, with different patterns for only the oleic and linoleic fatty acids. This indicates that the dietary fatty acid composition and their consumption by the chickens were different. Differences in fatty acid contents in animal products have been found to be affected by lipid metabolism and fats/fatty acids composition [12, 14, 18, 19, 48]. This agrees with the present results. However, there are also several other factors of a secondary order that can affect the lipid and cholesterol contents of meat, such as feed additive supplementation [13, 25, 27, 43], the age, sex and strain of the broilers [49], flock husbandry, the season, the length of the rearing period [50–52], and the cooking and storage conditions [21, 53].

Correlation analyses can draw the type and strength of the relationships among the different variables under investigation. SFA showed a significant strong negative relationship with the hypocholesterolemic index, but a moderate positive relationship with TAC, as SFA is less susceptible to lipid peroxidation. The correlation between UFA and the hypocholesterolemic index was significantly positive, while the relationship with the atherogenic and thrombotic indices was strongly negative. This can be attributed particularly to PUFA. PUFA exhibited a significantly positive relationship of a moderate magnitude with the hypocholesterolemic index and a significantly negative relationship of a moderate magnitude with total cholesterol, the atherogenic index, TAC and MDA. Thus meat enhanced with PUFA can have a beneficial health benefit for humans, as recently recommended and as shown by increasing consumer preferences [12, 18, 19].

The relationship between omega-3 and the thrombotic index and the hypocholesterolemic index was significantly positive with a moderate degree of power. In addition, omega-6 and total cholesterol, the atherogenic index, TAC and MDA showed negative correlations with moderate power, but positive relationships of a moderate magnitude with the hypocholesterolemic index. Thus omega-6 and omega-3 and their ratio (omega-6/omega-3) are the principle fatty acids controlling the hypocholesterolemic index, and thus should be given the highest priority in broiler feeding programs due to their positive health implications for human [12, 19, 47]. Omega-3 plays a major role in regulating the thrombotic index, while omega-6 is dominant in the atherogenic index, TAC and MDA [4]. The negative impact of omega-6 on TAC and MDA can be attributed to their high susceptibility to lipid peroxidation and thus low antioxidant status in animal products. Hence antioxidant supplementation is essential for diets containing increasing amounts of PUFA in animal products [13, 43]. A healthy animal product can be characterized by low hypercholesterolemic, atherogenic and thrombogenic indices [4, 33, 42].

Myristic and palmitic acids are among the most atherogenic agents, whereas stearic is thought to be neutral with respect to atherogenicity but is instead considered to be thrombogenic [33, 42]. Poultry meat with high UFA content is preferable for customers due to its low cholesterol (hypocholesterolemic index), LDL/HDL and lower atherogenic index [29, 30, 53]. In literature, feeding an atherogenic diet exhibited marked elevation in total serum cholesterol (hypercholesterolemic), LDL, VLDL, and triglyceride levels, along with decreased HDL levels [54]. Hence low atherogenic, thrombogenic and hypercholesterolemic foods are good for retarding atherosclerosis and thus the risk of cardiovascular disorders in human [4, 27, 28, 42]. Furthermore, animal products with low thrombogenicity decrease the threat of atrial fibrillation [55]. It should be mentioned that consumption of 300 g of broiler meat resulted in 216 mg of cholesterol, which represented (72%) of the recommended 300 mg daily allowance of cholesterol for adults [56].

## Conclusions

Fatty acids profiles and the hypocholesterolemic and atherogenic indices of broiler meat in the retail market in Jeddah city, Saudi Arabia during April, May and June 2014 showed significant differences, with the potential for favourable fatty acids to be boosted. Such variability indicates the needs for a feeding strategy to enhance the favourable fatty acids that may positively impact the health of the consumer, lowering the risk of hypercholesterolemia, atherosclerosis, and thrombogenesis although further studies are needed.

## Methods

### Materials

Ninety whole carcasses of chickens were randomly collected during April-May-June 2014 from 6 sources, named A, B, C, D, E and F, chosen randomly from a retail store. The samples represented two types of chicken carcasses, imported frozen carcasses (A, B and C) from Brazil and France, and locally produced fresh carcasses (D, E, and F), in the Hyper Panda retail market, in Jeddah, Saudi Arabia. Meat samples were 5-pooled samples/source/time, which were replicated 3 times, resulting in a total of 15 samples/source of each meat type. The carcasses were chosen from grade A of each source and were of similar carcass weight (1 kg). The chicken carcasses had similar dates of production and expiration when collected from the retail markets. The carcasses were cut into two halves. The right side of the carcasses was skinned and deboned, and the meat was minced using a meat mincer (Moulinex-HV8, France). The meat samples were pooled over time to represents 5 samples/source/type, and were immediately frozen at -20^ο^C until further analysed.

The chickens were reared under commercial operating conditions and were managed using common husbandry practice for broiler chickens, but the details of rearing and husbandry practices and the feeding regimens were not provided by the producing companies. The chickens were slaughtered in an automatic slaughterhouse according to the Islamic method.

### Measurements

#### Cholesterol profiles, total antioxidants capacity and malondialdehyde

Lipids were extracted according to [57] to determine meat cholesterol [58], high-density lipoprotein (HDL) [59] and low-density lipoprotein (LDL) [60]. Total antioxidant capacity (mmoles\ 100g fresh weight) and lipid peroxidation biomarkers, such as malonyldialdehyde (mmoles\g fresh weight), were determined according to [61, 62], respectively. The determinations were done using commercial kits (Diamond Diagnostics, 33 Fiske St, Holliston MA 01746, USA).

### Fatty acids profiles of the meat lipids

A part of the lipids extracted from the meat was used for the determination of fatty acid profiles of the meat by Shimadzu Gas Chromatograph GC-4CM (PFE) connected to a glass column (3X 3mm i.d.) packed with 5% diethylene glycol succinate (DEGS) and detector of flame ionization (FID). The separation and identification was as follows: Column temperature 1800C; temperature for both injector and detector were 2700C; nitrogen, hydrogen and air were used as a carrier gas at flow rates (ml/min.) of 30, 1 and 0.5, respectively, according to [63]. A standard of fatty acid methyl esters was treated under the same conditions before analysis of the samples. The fatty acids of the sample were identified by comparison of their retention times (tR) with the standard. The sample size was 5-pooled samples over 3 times per each meat source of each type of meat. Atherogenic and thrombogenic indexes were calculated according to [64]. The hypocholesterolemic index was calculated according to [65].

### Statistical analyses

Data were statistically analysed according to [66] using the PROC NESTED procedures according the following model:

$$ {\mathrm{Y}}_{\mathrm{i}\mathrm{j}\mathrm{k}} = \upmu + {\mathrm{C}}_{\mathrm{i}} + {\mathrm{B}}_{\mathrm{i}\mathrm{j}} + {\mathrm{e}}_{\mathrm{i}\mathrm{j}\mathrm{k}} $$

Y_ijk_ = observed value of the dependent variable; μ: overall mean; Ci: effect of meat type (frozen vs. fresh); Bij: effect of type within source; eijklm: experimental error of random distribution (0,σ2).

Before running the statistical analyses, all percentages were transformed to arc sins to normalize data distribution. Significance of differences was tested at (*P* < 0.05) for all means according to [67].
